# Bis(acetato-κ*O*)bis­(μ_3_-quinolin-8-olato-κ^4^ *N*,*O*:*O*:*O*)tetra­kis(μ_2_-quinolin-8-olato-κ^3^ *N*,*O*:*O*)tetra­zinc(II) dihydrate

**DOI:** 10.1107/S1600536809020157

**Published:** 2009-06-06

**Authors:** Elham Sattarzadeh, Gholamhossein Mohammadnezhad, Mostafa M. Amini, Seik Weng Ng

**Affiliations:** a Department of Chemistry, General Campus, Shahid Beheshti University, Tehran 1983963113, Iran; b Department of Chemistry, University of Malaya, 50603 Kuala Lumpur, Malaysia

## Abstract

In the centrosymmetric title compound, [Zn_4_(C_9_H_6_NO)_6_(C_2_H_3_O_2_)_2_]·2H_2_O, the Zn^II^ atom that is bonded to one O atom of the acetate group is chelated by a quinolin-8-olate anion. This Zn atom is also bonded to the oxide O atoms of two other quinolin-8-olate anions, which themselves engage in chelation to the other Zn^II^ atoms. The Zn^II^ atom is five-coordinate in a square-pyramidal coordination geometry. The second Zn^II^ atom is six-coordinate as it is linked to two oxide O atoms of the anions that chelate to the acetate-bound metal atom, and is chelated by two quinolin-8-olate ligands. The uncoordinated water mol­ecule is disordered over two positions in a 4:1 ratio. O—H⋯O hydrogen bonds between the water molecules and the free O atoms of the carboxylate groups consolidate the crystal packing.

## Related literature

For previous studies of the zinc derivatives of 8-hydroxy­quinoline, see: Sattarzadeh *et al.* (2009*a* ▶,*b* ▶).
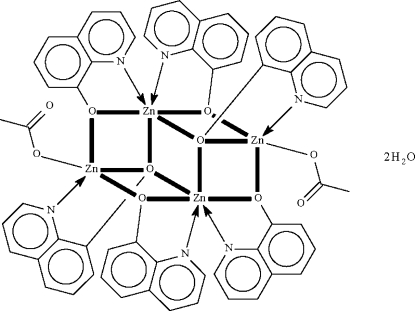


## Experimental

### 

#### Crystal data

[Zn_4_(C_9_H_6_NO)_6_(C_2_H_3_O_2_)_2_]·2H_2_O
*M* *_r_* = 1280.49
Triclinic, 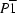

*a* = 11.3313 (3) Å
*b* = 11.8367 (3) Å
*c* = 13.5379 (3) Å
α = 111.070 (2)°
β = 107.740 (2)°
γ = 112.579 (2)°
*V* = 1342.16 (6) Å^3^
*Z* = 1
Mo *K*α radiation
μ = 1.84 mm^−1^
*T* = 100 K
0.15 × 0.10 × 0.05 mm

#### Data collection

Bruker SMART APEX diffractometer
Absorption correction: multi-scan (*SADABS*; Sheldrick, 1996 ▶) *T*_min_ = 0.657, *T*_max_ = 0.746 (expected range = 0.803–0.912)
12632 measured reflections
6146 independent reflections
4418 reflections with *I* > 2σ(*I*)
*R*_int_ = 0.037

#### Refinement

*R*[*F* ^2^ > 2σ(*F* ^2^)] = 0.039
*wR*(*F* ^2^) = 0.102
*S* = 1.00
6146 reflections
372 parameters
12 restraints
H-atom parameters constrained
Δρ_max_ = 0.63 e Å^−3^
Δρ_min_ = −0.44 e Å^−3^

### 

Data collection: *APEX2* (Bruker, 2008 ▶); cell refinement: *SAINT* (Bruker, 2008 ▶); data reduction: *SAINT*; program(s) used to solve structure: *SHELXS97* (Sheldrick, 2008 ▶); program(s) used to refine structure: *SHELXL97* (Sheldrick, 2008 ▶); molecular graphics: *X-SEED* (Barbour, 2001 ▶); software used to prepare material for publication: *publCIF* (Westrip, 2009 ▶).

## Supplementary Material

Crystal structure: contains datablocks global, I. DOI: 10.1107/S1600536809020157/tk2455sup1.cif

Structure factors: contains datablocks I. DOI: 10.1107/S1600536809020157/tk2455Isup2.hkl

Additional supplementary materials:  crystallographic information; 3D view; checkCIF report

## Figures and Tables

**Table 1:** Hydrogen-bond geometry (Å, °)

*D*—H⋯*A*	*D*—H	H⋯*A*	*D*⋯*A*	*D*—H⋯*A*
O1w—H1w1⋯O4	0.84	2.04	2.858 (6)	166
O1w’—H1w3⋯O4	0.84	2.04	2.83 (2)	157

**Fig. 1.**
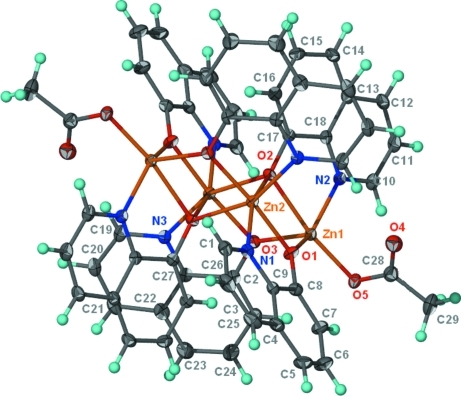
Thermal ellipsoid plot (Barbour, 2001) of [Zn2(C2H3O2)(C9H6NO)3]2.2H2O; ellipsoids are drawn at the 70% probability level and H atoms with an arbitrary radius. The disordered lattice water molecule is not shown.

## supplementary crystallographic information

### Experimental

Zinc acetate dihydrate (0.22 g, 0.1 mmol) and 2-methyl-8-hydroxyquinoline (0.14 g, 1 mmol) were loaded into a convection tube. The tube was filled with dry methanol and kept at 333 K. Crystals were collected from the side-arm after a week. The crystals are stable up to 573 K.

### Refinement

Carbon-bound H-atoms were placed in calculated positions (C–H 0.95 to 0.98 Å) and were included in the refinement in the riding model approximation with *U*(H) set to 1.2 to 1.5*U*(C).

The water O atom is disordered over two positions in a 4:1 ratio. The two pairs of H atoms were placed in chemically sensible positions on the basis that one of each set forms a hydrogen bond to an acceptor. The O–H distances were fixed at 0.84 Å, and their isotropic temperature factors were tied to those of the O atoms. The anisotropic displacement parameters of the disordered O atoms were restrained to be nearly isotropic.

### Crystal data

**Table:**

[Zn_4_(C_9_H_6_NO)_6_(C_2_H_3_O_2_)_2_]·2H_2_O	*Z* = 1
*M**_r_* = 1280.49	*F*(000) = 652
Triclinic, *P*1	*D*_x_ = 1.584 Mg m^−^^3^
Hall symbol: -P 1	Mo *K*α radiation, λ = 0.71073 Å
*a* = 11.3313 (3) Å	Cell parameters from 2730 reflections
*b* = 11.8367 (3) Å	θ = 2.3–26.7°
*c* = 13.5379 (3) Å	µ = 1.84 mm^−^^1^
α = 111.070 (2)°	*T* = 100 K
β = 107.740 (2)°	Prism, yellow
γ = 112.579 (2)°	0.15 × 0.10 × 0.05 mm
*V* = 1342.16 (6) Å^3^	

### Data collection

**Table:**

Bruker SMART APEX diffractometer	6146 independent reflections
Radiation source: fine-focus sealed tube	4418 reflections with *I* > 2σ(*I*)
graphite	*R*_int_ = 0.037
ω scans	θ_max_ = 27.5°, θ_min_ = 1.9°
Absorption correction: multi-scan (*SADABS*; Sheldrick, 1996)	*h* = −14→14
*T*_min_ = 0.657, *T*_max_ = 0.746	*k* = −15→15
12632 measured reflections	*l* = −17→16

### Refinement

**Table:**

Refinement on *F*^2^	Primary atom site location: structure-invariant direct methods
Least-squares matrix: full	Secondary atom site location: difference Fourier map
*R*[*F*^2^ > 2σ(*F*^2^)] = 0.039	Hydrogen site location: inferred from neighbouring sites
*wR*(*F*^2^) = 0.102	H-atom parameters constrained
*S* = 1.00	*w* = 1/[σ^2^(*F*_o_^2^) + (0.0512*P*)^2^] where *P* = (*F*_o_^2^ + 2*F*_c_^2^)/3
6146 reflections	(Δ/σ)_max_ = 0.001
372 parameters	Δρ_max_ = 0.63 e Å^−^^3^
12 restraints	Δρ_min_ = −0.44 e Å^−^^3^

### Special details

**Table:**

Geometry. All e.s.d.'s (except the e.s.d. in the dihedral angle between two l.s. planes) are estimated using the full covariance matrix. The cell e.s.d.'s are taken into account individually in the estimation of e.s.d.'s in distances, angles and torsion angles; correlations between e.s.d.'s in cell parameters are only used when they are defined by crystal symmetry. An approximate (isotropic) treatment of cell e.s.d.'s is used for estimating e.s.d.'s involving l.s. planes.

### Fractional atomic coordinates and isotropic or equivalent isotropic displacement parameters (Å^2^)

**Table:**

	*x*	*y*	*z*	*U*_iso_*/*U*_eq_	Occ. (<1)
Zn1	0.73984 (4)	0.30627 (4)	0.26111 (3)	0.01608 (10)	
Zn2	0.97306 (4)	0.63077 (4)	0.52018 (3)	0.01623 (10)	
O1	0.8598 (2)	0.5204 (2)	0.32955 (19)	0.0198 (5)	
O2	0.8541 (2)	0.4092 (2)	0.46797 (19)	0.0163 (4)	
O3	0.8952 (2)	0.2597 (2)	0.28947 (19)	0.0186 (5)	
O4	0.5060 (3)	0.3123 (3)	0.1537 (2)	0.0303 (6)	
O5	0.6110 (2)	0.2083 (2)	0.0791 (2)	0.0209 (5)	
O1W	0.5348 (9)	0.5762 (6)	0.1876 (6)	0.090 (3)	0.80 (2)
H1W1	0.5392	0.5080	0.1901	0.135*	0.80 (2)
H1W2	0.4513	0.5621	0.1737	0.135*	0.80 (2)
O1W'	0.463 (4)	0.542 (3)	0.223 (2)	0.092 (12)	0.20 (2)
H1W3	0.4545	0.4638	0.1826	0.139*	0.20 (2)
H1W4	0.4981	0.5977	0.2003	0.139*	0.20 (2)
N1	1.0559 (3)	0.8045 (3)	0.4946 (2)	0.0182 (6)	
N2	0.5909 (3)	0.1627 (3)	0.2853 (2)	0.0178 (6)	
N3	1.1687 (3)	0.3302 (3)	0.4247 (2)	0.0174 (6)	
C1	1.1537 (3)	0.9450 (4)	0.5797 (3)	0.0232 (7)	
H1A	1.1990	0.9756	0.6641	0.028*	
C2	1.1928 (4)	1.0508 (4)	0.5495 (3)	0.0276 (8)	
H2A	1.2638	1.1509	0.6124	0.033*	
C3	1.1272 (4)	1.0069 (4)	0.4285 (4)	0.0284 (8)	
H3	1.1520	1.0772	0.4069	0.034*	
C4	1.0221 (4)	0.8573 (4)	0.3345 (3)	0.0241 (7)	
C5	0.9514 (4)	0.8022 (4)	0.2072 (3)	0.0316 (8)	
H5	0.9698	0.8670	0.1795	0.038*	
C6	0.8564 (4)	0.6556 (4)	0.1240 (3)	0.0329 (9)	
H6	0.8110	0.6192	0.0383	0.040*	
C7	0.8239 (4)	0.5569 (4)	0.1620 (3)	0.0267 (8)	
H7	0.7578	0.4554	0.1016	0.032*	
C8	0.8863 (3)	0.6050 (3)	0.2849 (3)	0.0202 (7)	
C9	0.9897 (3)	0.7585 (3)	0.3730 (3)	0.0177 (6)	
C10	0.4661 (3)	0.0363 (4)	0.1931 (3)	0.0229 (7)	
H10	0.4467	0.0028	0.1112	0.028*	
C11	0.3616 (4)	−0.0494 (4)	0.2128 (3)	0.0258 (8)	
H11	0.2737	−0.1402	0.1449	0.031*	
C12	0.3862 (3)	−0.0027 (4)	0.3285 (3)	0.0234 (7)	
H12	0.3139	−0.0592	0.3416	0.028*	
C13	0.5199 (3)	0.1307 (3)	0.4303 (3)	0.0198 (7)	
C14	0.5559 (4)	0.1872 (4)	0.5534 (3)	0.0237 (7)	
H14	0.4878	0.1370	0.5730	0.028*	
C15	0.6902 (4)	0.3157 (4)	0.6457 (3)	0.0233 (7)	
H15	0.7142	0.3536	0.7290	0.028*	
C16	0.7934 (3)	0.3927 (3)	0.6193 (3)	0.0196 (7)	
H16	0.8864	0.4800	0.6852	0.024*	
C17	0.7617 (3)	0.3435 (3)	0.5006 (3)	0.0164 (6)	
C18	0.6216 (3)	0.2095 (3)	0.4029 (3)	0.0168 (6)	
C19	1.3027 (3)	0.3629 (3)	0.4935 (3)	0.0228 (7)	
H19	1.3424	0.4049	0.5797	0.027*	
C20	1.3880 (4)	0.3375 (3)	0.4430 (3)	0.0234 (7)	
H20	1.4818	0.3580	0.4943	0.028*	
C21	1.3372 (4)	0.2840 (4)	0.3216 (3)	0.0265 (8)	
H21	1.3965	0.2700	0.2881	0.032*	
C22	1.1942 (4)	0.2489 (4)	0.2443 (3)	0.0250 (7)	
C23	1.1331 (4)	0.1976 (5)	0.1187 (3)	0.0376 (10)	
H23	1.1877	0.1843	0.0795	0.045*	
C24	0.9941 (4)	0.1666 (5)	0.0524 (3)	0.0389 (10)	
H24	0.9531	0.1312	−0.0330	0.047*	
C25	0.9106 (4)	0.1857 (4)	0.1075 (3)	0.0282 (8)	
H25	0.8143	0.1625	0.0588	0.034*	
C26	0.9662 (3)	0.2371 (3)	0.2302 (3)	0.0197 (7)	
C27	1.1122 (3)	0.2711 (3)	0.3013 (3)	0.0181 (6)	
C28	0.5110 (4)	0.2375 (4)	0.0656 (3)	0.0255 (8)	
C29	0.3962 (4)	0.1727 (5)	−0.0659 (4)	0.0422 (10)	
H29A	0.3284	0.2048	−0.0657	0.063*	
H29B	0.3393	0.0662	−0.1102	0.063*	
H29C	0.4466	0.2050	−0.1070	0.063*	

### Atomic displacement parameters (Å^2^)

**Table:**

	*U*^11^	*U*^22^	*U*^33^	*U*^12^	*U*^13^	*U*^23^
Zn1	0.01469 (19)	0.01421 (19)	0.0145 (2)	0.00664 (16)	0.00615 (15)	0.00615 (16)
Zn2	0.01675 (19)	0.01366 (19)	0.0148 (2)	0.00730 (16)	0.00658 (16)	0.00720 (16)
O1	0.0218 (11)	0.0119 (11)	0.0143 (11)	0.0041 (9)	0.0047 (9)	0.0067 (9)
O2	0.0158 (11)	0.0131 (11)	0.0192 (12)	0.0064 (9)	0.0102 (9)	0.0090 (9)
O3	0.0176 (11)	0.0180 (11)	0.0161 (12)	0.0093 (10)	0.0085 (9)	0.0065 (10)
O4	0.0328 (14)	0.0316 (14)	0.0345 (15)	0.0214 (12)	0.0194 (12)	0.0191 (12)
O5	0.0185 (11)	0.0206 (12)	0.0161 (12)	0.0101 (10)	0.0059 (9)	0.0071 (10)
O1W	0.094 (5)	0.061 (3)	0.112 (5)	0.056 (3)	0.039 (3)	0.041 (3)
O1W'	0.099 (15)	0.090 (13)	0.093 (14)	0.065 (11)	0.030 (7)	0.056 (9)
N1	0.0178 (13)	0.0154 (13)	0.0205 (14)	0.0095 (12)	0.0101 (12)	0.0084 (12)
N2	0.0173 (13)	0.0158 (13)	0.0177 (14)	0.0084 (11)	0.0081 (11)	0.0085 (12)
N3	0.0183 (13)	0.0129 (13)	0.0205 (14)	0.0084 (11)	0.0087 (12)	0.0099 (12)
C1	0.0205 (17)	0.0201 (17)	0.0247 (18)	0.0102 (15)	0.0117 (15)	0.0096 (15)
C2	0.0243 (18)	0.0160 (17)	0.039 (2)	0.0092 (15)	0.0188 (17)	0.0115 (16)
C3	0.0301 (19)	0.0231 (18)	0.046 (2)	0.0149 (16)	0.0276 (18)	0.0240 (18)
C4	0.0244 (17)	0.0251 (18)	0.034 (2)	0.0146 (15)	0.0196 (16)	0.0211 (17)
C5	0.039 (2)	0.037 (2)	0.034 (2)	0.0209 (19)	0.0246 (18)	0.0283 (19)
C6	0.039 (2)	0.037 (2)	0.023 (2)	0.0191 (19)	0.0151 (17)	0.0194 (18)
C7	0.0272 (18)	0.0253 (18)	0.0187 (18)	0.0090 (16)	0.0091 (15)	0.0124 (15)
C8	0.0214 (16)	0.0189 (16)	0.0221 (18)	0.0107 (14)	0.0125 (14)	0.0123 (15)
C9	0.0174 (15)	0.0175 (16)	0.0203 (17)	0.0107 (13)	0.0107 (14)	0.0100 (14)
C10	0.0193 (16)	0.0185 (17)	0.0210 (18)	0.0088 (14)	0.0048 (14)	0.0084 (14)
C11	0.0168 (16)	0.0160 (17)	0.029 (2)	0.0034 (14)	0.0040 (15)	0.0113 (15)
C12	0.0173 (16)	0.0220 (17)	0.035 (2)	0.0096 (14)	0.0136 (15)	0.0198 (16)
C13	0.0175 (16)	0.0202 (17)	0.0267 (19)	0.0114 (14)	0.0122 (14)	0.0155 (15)
C14	0.0224 (17)	0.0262 (18)	0.033 (2)	0.0139 (15)	0.0180 (16)	0.0213 (17)
C15	0.0298 (18)	0.0265 (18)	0.0239 (18)	0.0179 (16)	0.0168 (16)	0.0178 (16)
C16	0.0210 (16)	0.0167 (16)	0.0209 (17)	0.0102 (14)	0.0105 (14)	0.0103 (14)
C17	0.0195 (16)	0.0153 (15)	0.0210 (17)	0.0122 (14)	0.0114 (14)	0.0119 (14)
C18	0.0175 (15)	0.0154 (15)	0.0187 (17)	0.0110 (13)	0.0092 (13)	0.0084 (13)
C19	0.0178 (16)	0.0144 (16)	0.0244 (18)	0.0056 (14)	0.0040 (14)	0.0098 (14)
C20	0.0187 (16)	0.0166 (16)	0.034 (2)	0.0112 (14)	0.0107 (15)	0.0143 (15)
C21	0.0226 (17)	0.0216 (17)	0.036 (2)	0.0138 (15)	0.0168 (16)	0.0129 (16)
C22	0.0260 (18)	0.0200 (17)	0.029 (2)	0.0143 (15)	0.0157 (16)	0.0109 (15)
C23	0.035 (2)	0.053 (3)	0.029 (2)	0.029 (2)	0.0220 (19)	0.017 (2)
C24	0.033 (2)	0.058 (3)	0.019 (2)	0.026 (2)	0.0135 (17)	0.0129 (19)
C25	0.0234 (18)	0.036 (2)	0.0217 (19)	0.0177 (17)	0.0110 (15)	0.0113 (17)
C26	0.0194 (16)	0.0146 (16)	0.0211 (18)	0.0089 (14)	0.0096 (14)	0.0076 (14)
C27	0.0197 (16)	0.0125 (15)	0.0198 (17)	0.0086 (13)	0.0092 (14)	0.0079 (13)
C28	0.0226 (17)	0.0231 (18)	0.0262 (19)	0.0104 (15)	0.0105 (15)	0.0137 (16)
C29	0.031 (2)	0.050 (3)	0.035 (2)	0.023 (2)	0.0066 (18)	0.022 (2)

### Geometric parameters (Å, °)

**Table:**

Zn1—O5	2.000 (2)	C5—C6	1.364 (5)
Zn1—O1	2.007 (2)	C5—H5	0.9500
Zn1—O3	2.009 (2)	C6—C7	1.407 (5)
Zn1—N2	2.093 (3)	C6—H6	0.9500
Zn1—O2	2.265 (2)	C7—C8	1.374 (4)
Zn2—O1	2.070 (2)	C7—H7	0.9500
Zn2—O3^i^	2.078 (2)	C8—C9	1.433 (4)
Zn2—N3^i^	2.098 (3)	C10—C11	1.402 (5)
Zn2—N1	2.111 (3)	C10—H10	0.9500
Zn2—O2	2.137 (2)	C11—C12	1.353 (5)
Zn2—O2^i^	2.158 (2)	C11—H11	0.9500
O1—C8	1.331 (4)	C12—C13	1.419 (4)
O2—C17	1.341 (3)	C12—H12	0.9500
O2—Zn2^i^	2.158 (2)	C13—C14	1.403 (5)
O3—C26	1.329 (4)	C13—C18	1.421 (4)
O3—Zn2^i^	2.078 (2)	C14—C15	1.374 (5)
O4—C28	1.236 (4)	C14—H14	0.9500
O5—C28	1.288 (4)	C15—C16	1.412 (4)
O1W—H1W1	0.8399	C15—H15	0.9500
O1W—H1W2	0.8400	C16—C17	1.367 (4)
O1W—H1W3	1.2538	C16—H16	0.9500
O1W—H1W4	0.6012	C17—C18	1.437 (4)
O1W'—H1W1	1.2197	C19—C20	1.404 (5)
O1W'—H1W2	0.7744	C19—H19	0.9500
O1W'—H1W3	0.8400	C20—C21	1.352 (5)
O1W'—H1W4	0.8400	C20—H20	0.9500
N1—C1	1.322 (4)	C21—C22	1.425 (5)
N1—C9	1.364 (4)	C21—H21	0.9500
N2—C10	1.324 (4)	C22—C23	1.399 (5)
N2—C18	1.357 (4)	C22—C27	1.416 (4)
N3—C19	1.323 (4)	C23—C24	1.371 (5)
N3—C27	1.362 (4)	C23—H23	0.9500
N3—Zn2^i^	2.098 (3)	C24—C25	1.406 (5)
C1—C2	1.408 (5)	C24—H24	0.9500
C1—H1A	0.9500	C25—C26	1.367 (5)
C2—C3	1.363 (5)	C25—H25	0.9500
C2—H2A	0.9500	C26—C27	1.438 (4)
C3—C4	1.418 (5)	C28—C29	1.514 (5)
C3—H3	0.9500	C29—H29A	0.9800
C4—C5	1.409 (5)	C29—H29B	0.9800
C4—C9	1.414 (4)	C29—H29C	0.9800
			
O5—Zn1—O1	106.13 (9)	O1—C8—C7	125.4 (3)
O5—Zn1—O3	107.95 (9)	O1—C8—C9	116.9 (3)
O1—Zn1—O3	102.85 (9)	C7—C8—C9	117.7 (3)
O5—Zn1—N2	97.79 (9)	N1—C9—C4	121.8 (3)
O1—Zn1—N2	138.81 (10)	N1—C9—C8	117.3 (3)
O3—Zn1—N2	100.90 (9)	C4—C9—C8	121.0 (3)
O5—Zn1—O2	171.45 (8)	N2—C10—C11	121.9 (3)
O1—Zn1—O2	76.48 (8)	N2—C10—H10	119.1
O3—Zn1—O2	79.00 (8)	C11—C10—H10	119.1
N2—Zn1—O2	75.65 (9)	C12—C11—C10	120.0 (3)
O1—Zn2—O3^i^	174.06 (8)	C12—C11—H11	120.0
O1—Zn2—N3^i^	106.56 (9)	C10—C11—H11	120.0
O3^i^—Zn2—N3^i^	79.38 (9)	C11—C12—C13	120.0 (3)
O1—Zn2—N1	79.00 (9)	C11—C12—H12	120.0
O3^i^—Zn2—N1	100.41 (9)	C13—C12—H12	120.0
N3^i^—Zn2—N1	97.67 (10)	C14—C13—C12	124.1 (3)
O1—Zn2—O2	78.13 (8)	C14—C13—C18	119.3 (3)
O3^i^—Zn2—O2	101.96 (8)	C12—C13—C18	116.6 (3)
N3^i^—Zn2—O2	92.21 (9)	C15—C14—C13	119.5 (3)
N1—Zn2—O2	156.85 (9)	C15—C14—H14	120.2
O1—Zn2—O2^i^	94.12 (8)	C13—C14—H14	120.2
O3^i^—Zn2—O2^i^	80.07 (8)	C14—C15—C16	121.4 (3)
N3^i^—Zn2—O2^i^	156.41 (9)	C14—C15—H15	119.3
N1—Zn2—O2^i^	97.33 (8)	C16—C15—H15	119.3
O2—Zn2—O2^i^	80.84 (8)	C17—C16—C15	121.1 (3)
C8—O1—Zn1	137.0 (2)	C17—C16—H16	119.5
C8—O1—Zn2	114.79 (19)	C15—C16—H16	119.5
Zn1—O1—Zn2	108.21 (9)	O2—C17—C16	124.7 (3)
C17—O2—Zn2	130.34 (18)	O2—C17—C18	117.0 (3)
C17—O2—Zn2^i^	116.92 (17)	C16—C17—C18	118.3 (3)
Zn2—O2—Zn2^i^	99.16 (8)	N2—C18—C13	122.0 (3)
C17—O2—Zn1	111.59 (18)	N2—C18—C17	117.6 (3)
Zn2—O2—Zn1	97.19 (8)	C13—C18—C17	120.3 (3)
Zn2^i^—O2—Zn1	94.70 (8)	N3—C19—C20	122.0 (3)
C26—O3—Zn1	132.98 (19)	N3—C19—H19	119.0
C26—O3—Zn2^i^	113.58 (18)	C20—C19—H19	119.0
Zn1—O3—Zn2^i^	105.54 (9)	C21—C20—C19	120.3 (3)
C28—O5—Zn1	105.6 (2)	C21—C20—H20	119.9
H1W1—O1W—H1W2	110.6	C19—C20—H20	119.9
H1W3—O1W'—H1W4	108.9	C20—C21—C22	119.6 (3)
C1—N1—C9	119.7 (3)	C20—C21—H21	120.2
C1—N1—Zn2	128.3 (2)	C22—C21—H21	120.2
C9—N1—Zn2	111.9 (2)	C23—C22—C27	119.2 (3)
C10—N2—C18	119.5 (3)	C23—C22—C21	124.3 (3)
C10—N2—Zn1	123.8 (2)	C27—C22—C21	116.6 (3)
C18—N2—Zn1	116.6 (2)	C24—C23—C22	119.6 (3)
C19—N3—C27	119.0 (3)	C24—C23—H23	120.2
C19—N3—Zn2^i^	128.9 (2)	C22—C23—H23	120.2
C27—N3—Zn2^i^	111.93 (19)	C23—C24—C25	121.7 (3)
N1—C1—C2	122.3 (3)	C23—C24—H24	119.1
N1—C1—H1A	118.8	C25—C24—H24	119.1
C2—C1—H1A	118.8	C26—C25—C24	120.8 (3)
C3—C2—C1	118.8 (3)	C26—C25—H25	119.6
C3—C2—H2A	120.6	C24—C25—H25	119.6
C1—C2—H2A	120.6	O3—C26—C25	124.5 (3)
C2—C3—C4	120.6 (3)	O3—C26—C27	117.3 (3)
C2—C3—H3	119.7	C25—C26—C27	118.2 (3)
C4—C3—H3	119.7	N3—C27—C22	122.4 (3)
C5—C4—C9	118.9 (3)	N3—C27—C26	117.1 (3)
C5—C4—C3	124.3 (3)	C22—C27—C26	120.5 (3)
C9—C4—C3	116.8 (3)	O4—C28—O5	122.9 (3)
C6—C5—C4	119.7 (3)	O4—C28—C29	121.1 (3)
C6—C5—H5	120.2	O5—C28—C29	116.0 (3)
C4—C5—H5	120.2	C28—C29—H29A	109.5
C5—C6—C7	121.6 (3)	C28—C29—H29B	109.5
C5—C6—H6	119.2	H29A—C29—H29B	109.5
C7—C6—H6	119.2	C28—C29—H29C	109.5
C8—C7—C6	121.1 (3)	H29A—C29—H29C	109.5
C8—C7—H7	119.4	H29B—C29—H29C	109.5
C6—C7—H7	119.4		
			
O5—Zn1—O1—C8	−10.2 (3)	Zn1—O1—C8—C7	2.4 (5)
O3—Zn1—O1—C8	103.1 (3)	Zn2—O1—C8—C7	−179.7 (3)
N2—Zn1—O1—C8	−133.2 (3)	Zn1—O1—C8—C9	−177.0 (2)
O2—Zn1—O1—C8	178.2 (3)	Zn2—O1—C8—C9	0.9 (3)
O5—Zn1—O1—Zn2	171.85 (9)	C6—C7—C8—O1	178.4 (3)
O3—Zn1—O1—Zn2	−74.88 (11)	C6—C7—C8—C9	−2.2 (5)
N2—Zn1—O1—Zn2	48.84 (17)	C1—N1—C9—C4	−0.2 (4)
O2—Zn1—O1—Zn2	0.27 (9)	Zn2—N1—C9—C4	176.6 (2)
N3^i^—Zn2—O1—C8	92.5 (2)	C1—N1—C9—C8	179.1 (3)
N1—Zn2—O1—C8	−2.3 (2)	Zn2—N1—C9—C8	−4.1 (3)
O2—Zn2—O1—C8	−178.7 (2)	C5—C4—C9—N1	179.0 (3)
O2^i^—Zn2—O1—C8	−99.0 (2)	C3—C4—C9—N1	0.0 (4)
N3^i^—Zn2—O1—Zn1	−89.06 (11)	C5—C4—C9—C8	−0.3 (5)
N1—Zn2—O1—Zn1	176.12 (12)	C3—C4—C9—C8	−179.3 (3)
O2—Zn2—O1—Zn1	−0.29 (9)	O1—C8—C9—N1	2.3 (4)
O2^i^—Zn2—O1—Zn1	79.45 (10)	C7—C8—C9—N1	−177.2 (3)
O1—Zn2—O2—C17	−126.2 (2)	O1—C8—C9—C4	−178.4 (3)
O3^i^—Zn2—O2—C17	59.9 (2)	C7—C8—C9—C4	2.1 (5)
N3^i^—Zn2—O2—C17	−19.7 (2)	C18—N2—C10—C11	1.9 (5)
N1—Zn2—O2—C17	−135.2 (3)	Zn1—N2—C10—C11	−173.0 (2)
O2^i^—Zn2—O2—C17	137.6 (3)	N2—C10—C11—C12	0.9 (5)
O1—Zn2—O2—Zn2^i^	96.21 (9)	C10—C11—C12—C13	−2.1 (5)
O3^i^—Zn2—O2—Zn2^i^	−77.72 (9)	C11—C12—C13—C14	−179.3 (3)
N3^i^—Zn2—O2—Zn2^i^	−157.33 (10)	C11—C12—C13—C18	0.7 (4)
N1—Zn2—O2—Zn2^i^	87.2 (2)	C12—C13—C14—C15	178.4 (3)
O2^i^—Zn2—O2—Zn2^i^	0.0	C18—C13—C14—C15	−1.7 (5)
O1—Zn2—O2—Zn1	0.24 (8)	C13—C14—C15—C16	0.0 (5)
O3^i^—Zn2—O2—Zn1	−173.69 (7)	C14—C15—C16—C17	1.5 (5)
N3^i^—Zn2—O2—Zn1	106.71 (9)	Zn2—O2—C17—C16	−50.7 (4)
N1—Zn2—O2—Zn1	−8.7 (2)	Zn2^i^—O2—C17—C16	81.0 (3)
O2^i^—Zn2—O2—Zn1	−95.97 (9)	Zn1—O2—C17—C16	−171.5 (2)
O1—Zn1—O2—C17	138.47 (19)	Zn2—O2—C17—C18	131.3 (2)
O3—Zn1—O2—C17	−115.28 (19)	Zn2^i^—O2—C17—C18	−96.9 (3)
N2—Zn1—O2—C17	−10.89 (18)	Zn1—O2—C17—C18	10.5 (3)
O1—Zn1—O2—Zn2	−0.25 (8)	C15—C16—C17—O2	−179.2 (3)
O3—Zn1—O2—Zn2	105.99 (9)	C15—C16—C17—C18	−1.2 (4)
N2—Zn1—O2—Zn2	−149.61 (10)	C10—N2—C18—C13	−3.4 (4)
O1—Zn1—O2—Zn2^i^	−100.12 (9)	Zn1—N2—C18—C13	171.9 (2)
O3—Zn1—O2—Zn2^i^	6.13 (8)	C10—N2—C18—C17	176.8 (3)
N2—Zn1—O2—Zn2^i^	110.52 (9)	Zn1—N2—C18—C17	−7.9 (3)
O5—Zn1—O3—C26	32.8 (3)	C14—C13—C18—N2	−177.9 (3)
O1—Zn1—O3—C26	−79.1 (3)	C12—C13—C18—N2	2.1 (4)
N2—Zn1—O3—C26	134.8 (3)	C14—C13—C18—C17	1.9 (4)
O2—Zn1—O3—C26	−152.3 (3)	C12—C13—C18—C17	−178.2 (3)
O5—Zn1—O3—Zn2^i^	178.57 (9)	O2—C17—C18—N2	−2.5 (4)
O1—Zn1—O3—Zn2^i^	66.64 (11)	C16—C17—C18—N2	179.4 (3)
N2—Zn1—O3—Zn2^i^	−79.45 (11)	O2—C17—C18—C13	177.7 (3)
O2—Zn1—O3—Zn2^i^	−6.58 (8)	C16—C17—C18—C13	−0.4 (4)
O1—Zn1—O5—C28	−72.3 (2)	C27—N3—C19—C20	−0.5 (5)
O3—Zn1—O5—C28	178.01 (19)	Zn2^i^—N3—C19—C20	−175.8 (2)
N2—Zn1—O5—C28	73.8 (2)	N3—C19—C20—C21	3.0 (5)
O1—Zn2—N1—C1	179.9 (3)	C19—C20—C21—C22	−2.1 (5)
O3^i^—Zn2—N1—C1	−6.2 (3)	C20—C21—C22—C23	177.8 (3)
N3^i^—Zn2—N1—C1	74.4 (3)	C20—C21—C22—C27	−0.9 (5)
O2—Zn2—N1—C1	−171.2 (2)	C27—C22—C23—C24	−1.5 (6)
O2^i^—Zn2—N1—C1	−87.4 (3)	C21—C22—C23—C24	179.8 (4)
O1—Zn2—N1—C9	3.41 (19)	C22—C23—C24—C25	0.3 (7)
O3^i^—Zn2—N1—C9	177.39 (19)	C23—C24—C25—C26	0.4 (6)
N3^i^—Zn2—N1—C9	−102.1 (2)	Zn1—O3—C26—C25	−27.0 (5)
O2—Zn2—N1—C9	12.4 (3)	Zn2^i^—O3—C26—C25	−170.7 (3)
O2^i^—Zn2—N1—C9	96.2 (2)	Zn1—O3—C26—C27	152.3 (2)
O5—Zn1—N2—C10	10.5 (3)	Zn2^i^—O3—C26—C27	8.6 (3)
O1—Zn1—N2—C10	136.1 (2)	C24—C25—C26—O3	179.3 (3)
O3—Zn1—N2—C10	−99.5 (2)	C24—C25—C26—C27	0.0 (5)
O2—Zn1—N2—C10	−175.1 (3)	C19—N3—C27—C22	−2.7 (4)
O5—Zn1—N2—C18	−164.5 (2)	Zn2^i^—N3—C27—C22	173.4 (2)
O1—Zn1—N2—C18	−38.9 (3)	C19—N3—C27—C26	179.9 (3)
O3—Zn1—N2—C18	85.4 (2)	Zn2^i^—N3—C27—C26	−4.0 (3)
O2—Zn1—N2—C18	9.9 (2)	C23—C22—C27—N3	−175.4 (3)
C9—N1—C1—C2	0.0 (5)	C21—C22—C27—N3	3.4 (5)
Zn2—N1—C1—C2	−176.2 (2)	C23—C22—C27—C26	1.9 (5)
N1—C1—C2—C3	0.3 (5)	C21—C22—C27—C26	−179.3 (3)
C1—C2—C3—C4	−0.5 (5)	O3—C26—C27—N3	−3.1 (4)
C2—C3—C4—C5	−178.6 (3)	C25—C26—C27—N3	176.3 (3)
C2—C3—C4—C9	0.3 (5)	O3—C26—C27—C22	179.5 (3)
C9—C4—C5—C6	−1.5 (5)	C25—C26—C27—C22	−1.2 (5)
C3—C4—C5—C6	177.4 (3)	Zn1—O5—C28—O4	0.2 (4)
C4—C5—C6—C7	1.5 (6)	Zn1—O5—C28—C29	−179.0 (2)
C5—C6—C7—C8	0.4 (6)		

Symmetry codes: (i) −*x*+2, −*y*+1, −*z*+1.

### Hydrogen-bond geometry (Å, °)

**Table:**

*D*—H···*A*	*D*—H	H···*A*	*D*···*A*	*D*—H···*A*
O1w—H1w1···O4	0.84	2.04	2.858 (6)	166
O1w'—H1w3···O4	0.84	2.04	2.83 (2)	157
